# Global glucose metabolism rate as diagnostic marker for disorder of consciousness of patients: quantitative FDG-PET study

**DOI:** 10.3389/fneur.2024.1425271

**Published:** 2025-01-03

**Authors:** Dongsheng Liu, Nan Wang, Ming Song, Xiaoke Chai, Qiheng He, Tianqing Cao, Dawei Kong, Zhuhuan Song, Guangming Zhang, Lei Liu, Xiaosong Wang, Guoqiang Chen, Shaoya Yin, Yi Yang, Jizong Zhao

**Affiliations:** ^1^Clinical College of Neurology, Neurosurgery and Neurorehabilitation, Tianjin Medical University, Tianjin, China; ^2^Department of Neurosurgery, Tianjin Huanhu Hospital, Tianjin, China; ^3^Department of Neurosurgery, Aviation General Hospital, Beijing, China; ^4^Department of Neurosurgery, Beijing Tiantan Hospital, Capital Medical University, Beijing, China; ^5^Department of Neurosurgery, Peking Union Medical College Hospital, Chinese Academy of Medical Sciences and Peking Union Medical College, Beijing, China; ^6^China National Clinical Research Center for Neurological Diseases, Beijing, China; ^7^National Laboratory of Pattern Recognition, Institute of Automation, The Chinese Academy of Sciences, Beijing, China; ^8^Brainnetome Center, Institute of Automation, The Chinese Academy of Sciences, Beijing, China; ^9^University of the Chinese Academy of Sciences, Beijing, China; ^10^Tianjin Key Laboratory of Cerebral Vascular and Neurodegenerative Diseases, Tianjin Neurosurgical Institute, Tianjin Huanhu Hospital, Tianjin, China; ^11^Brain-Computer Interface Transitional Research Center, Beijing Tiantan Hospital, Capital Medical University, Beijing, China; ^12^China National Center for Neurological Disorders, Beijing, China; ^13^National Research Center for Rehabilitation Technical Aids, Beijing, China; ^14^Chinese Institute for Brain Research Beijing, Beijing, China; ^15^Beijing Institute of Brain Disorders, Beijing, China; ^16^Nuffield Department of Clinical Neurosciences, University of Oxford, Oxford, United Kingdom

**Keywords:** FDG-PET, disorders of consciousness, global glucose metabolism rate, diagnostic marker, CRS-R

## Abstract

**Objective:**

This study was to employ 18F-flurodeoxyglucose (FDG-PET) to evaluate the resting-state brain glucose metabolism in a sample of 46 patients diagnosed with disorders of consciousness (DoC). The aim was to identify objective quantitative metabolic indicators and predictors that could potentially indicate the level of awareness in these patients.

**Methods:**

A cohort of 46 patients underwent Coma Recovery Scale-Revised (CRS-R) assessments in order to distinguish between the minimally conscious state (MCS) and the unresponsive wakefulness syndrome (UWS). Additionally, resting-state FDG-PET data were acquired from both the patient group and a control group consisting of 10 healthy individuals. The FDG-PET data underwent reorientation, spatial normalization to a stereotaxic space, and smoothing. The normalization procedure utilized a customized template following the methodology outlined by Phillips et al. Mean cortical metabolism of the overall sample was utilized for distinguishing between UWS and MCS, as well as for predicting the outcome at a 1-year follow-up through the application of receiver operating characteristic (ROC) analysis.

**Results:**

We used Global Glucose Metabolism as the Diagnostic Marker. A one-way ANOVA revealed that there was a statistically significant difference in cortical metabolic index between two groups (*F*(2, 53) = 7.26, *p* < 0.001). Multiple comparisons found that the mean of cortical metabolic index was significantly different between MCS (*M* = 4.19, *SD* = 0.64) and UWS group (*M* = 2.74, *SD* = 0.94*,p* < 0.001). Also, the mean of cortical metabolic index was significantly different between MCS and healthy group (*M* = 7.88, *SD* = 0.80,*p* < 0.001). Using the above diagnostic criterion, the diagnostic accuracy yielded an area under the curve (AUC) of 0.89 across the pooled cohort (95%CI 0.79–0.99). There was an 85% correct classification between MCS and UWS, with 88% sensitivity and 81% specificity for MCS. The best classification rate in the derivation cohort was achieved at a metabolic index of 3.32 (41% of the mean cortical metabolic index in healthy controls).

**Conclusion:**

Our findings demonstrate that conscious awareness requires a minimum of 41% of normal cortical activity, as indicated by metabolic rates.

## 1 Introduction

Minimal conscious disorder (MCS) is distinguished from the vegetative state/unresponsive wakefulness syndrome (*VS*/UWS) by the behavioral manifestations associated with consciousness, and this distinction between MCS and *VS* poses a significant challenge for clinicians responsible for managing patients with disorders of consciousness (DoC). Coma Recovery Scale-Revised (CRS-R), is regarded as the reference standard for clinical assessment of consciousness and is supported by international guidelines (1, 2). However, even with careful behavioral testing, the potential for misdiagnosis remains (3). Prior research has indicated that a range of 37 to 43% of patients who have been diagnosed with *VS* exhibit indications of consciousness. The misdiagnosis of such patients can have severe repercussions, particularly when it comes to making decisions regarding end-of-life care (4, 5). Clinical consensus methods have been found to misdiagnose patients with unresponsive wakefulness syndrome at a rate as high as 40% (5–7).

Neuroimaging techniques are currently being developed that are important for exploring the presence of brain activity indicated by patient consciousness, such as functional magnetic resonance imaging (fMRI), positron emission tomography (PET), electroencephalogram (EEG), and functional near-infrared spectroscopy (fNIRS) (8). These imaging techniques provide objectivity in the assessment of cerebral awareness, both in terms of intrinsic brain activity during periods of rest and in terms of specific cognitive responses to cerebral tasks. It is important to note that some individuals diagnosed with unresponsive arousal syndrome may possess the ability to consciously regulate their thoughts, thus implying the presence of at least some degree of consciousness (3, 9, 10). The identification and assessment of cognitive motor dissociation (CMD) present significant challenges in DoC. CMD patients retain intrinsic awareness but are unable to communicate it due to motor or communication impairments. This limitation exposes the inadequacies of conventional clinical evaluations, particularly when patients appear unresponsive. Accurate CMD diagnosis requires not only behavioral assessments but also objective biomarkers from advanced neuroimaging modalities (11). Functional neuroimaging techniques, including PET and fMRI, are pivotal in detecting CMD. These methods provide critical insights into brain activity and connectivity patterns, offering evidence of residual consciousness. For instance, Owen et al. demonstrated that some vegetative state patients exhibited fMRI-detected brain activity akin to healthy individuals during mental imagery tasks, indicating retained consciousness (9). Furthermore, studies have revealed diverse connectivity patterns across brain regions in CMD patients, underscoring the necessity of imaging for precise diagnosis and prognosis (12). In addition, 18F-FDG PET imaging can be used to help confirm the diagnosis of cognitive-motor dissociation, and an accurate diagnosis can help in the development of a long-term neurorehabilitation program, which can help to improve the patient’s prognosis (13).

Clinical use of PET-CT in China began in September 2002 (14). Resting-state 18F-flurodeoxyglucose (FDG-PET) assessment appears to have greater sensitivity in assisting in the clinical diagnosis of patients with DoC compared to fMRI (15). FDG-PET studies have confirmed the importance of the fronto-parietal network as it relates to consciousness, as evidenced by correlations between behavioral metrics and metabolic retention within this network. It has been shown that patients in a vegetative state who exhibit some degree of frontoparietal metabolic protection tend to have more favorable treatment outcomes compared to patients with low frontoparietal metabolism. FDG-PET shows promise in improving the clinical diagnosis of DoCs, but previous research has predominantly utilized intricate analytical approaches to distinguish between patients in MCS and those in unresponsive wakefulness states UWS. The current methods lack quantitative accuracy and are constrained by the constraints of visually guided assessments of changes in brain metabolism (15). In the present study, we conducted a cross-sectional multimodal investigation to quantify all glucose metabolic rate as a diagnostic indicator of patients with disorders of consciousness in order to explore the differences in prognosis and characteristic regional brain metabolism between patients with *VS*/UWS and those with MCS.

The preservation of specific behavioral or perceptual functions in patients with MCS is correlated with relative regional variations in metabolism, providing evidence for the assertion that network activations lead to localized increases in energy metabolism even in DoCs (16–18). The correlation between the functionality of distinct brain regions or networks and indicators of glucose metabolism holds more importance in gauging the extent of consciousness and is pivotal in the restoration of consciousness (19). Our hypothesis posits that the metabolic activity of allo-glucose serves as a predictor for the maintenance of conscious awareness, while variations in regional activity indicate the preservation of specific perceptual or cognitive functions. Our study aimed to assess the hypothesis regarding the preservation or recovery of consciousness following a 1-year follow-up through the measurement of overall brain metabolic rate. Utilizing resting-state PET scans, we analyzed patients with disorders of consciousness stemming from various etiologies such as traumatic brain injury, ischemic hypoxic encephalopathy, cerebral hemorrhage, and brainstem hemorrhage (20, 21).

## 2 Materials and methods

### 2.1 Participants

We retrospectively studied the clinical data of patients with DoC admitted to Beijing Tiantan Hospital of Capital Medical University between 2021 and 2023. Inclusion criteria: patients aged 18 ~ 60 years old who met the diagnosis of DoC and had undergone FDG-PET. Exclusion criteria: Patients under the age of 16, acute patients within 28 days of onset at the time of the PET scan, patients with fewer than five CRS-R assessments conducted within a specified time frame [as this is the recommended number for an accurate diagnosis (22)], patients diagnosed with coma or emergence from MCS, or those with an unclear diagnosis, were excluded from our analysis performed on the FDG-PET data we collected. 46 patients were finally identified for inclusion in the experimental group.

The 46 patients in the experimental group were patients between the ages of 18 and 57 years (including 27 males). The 46 patients included 21 patients with traumatic brain injury, 8 patients with hypoxic–ischemic encephalopathy, 10 patients with supratentorial intracerebral hemorrhage, and 7 patients with brainstem hemorrhage. All 46 patients underwent PET/CT within 2 to 4 months after the onset of the disease (Table 1). Another 10 healthy controls, aged 19 ~ 70 years, including 5 females, were selected.

**Table 1:** Patient demographics.

Study cohort	CRS-R diagnosis	*n*	Mean age ± SD	Male	Number of separate cases by etiology*
Derivation	UWS	10	37.9 ± 7.7	4	5–4–0-1
	MCS	13	40.3 ± 8.2	6	8–0–2-3
Validation	UWS	11	41.0 ± 8.7	9	3–4–4-0
	MCS	12	38.9 ± 11.4	8	5–0–4-3
	CON	10	39.1 ± 8.2	5	——
	total	56	39.5	32	21–8–10-7-10(con)

^*^The order of etiology: (traumatic brain injury)-(hypoxic–ischemic encephalopathy)-(supratentorial intracerebral hemorrhage)-(brainstem hemorrhage).

The procedure to split the population in derivation/validation sets is based on the enrollment time, and among patients enrolled earlier, 23 patients entered derivation after exclusion criteria. Using the same approach, an additional 23 patients were enrolled in validation.

The study was approved by the Biology Research Ethics Review Committee of the Institute of Automation, Chinese Academy of Sciences (IA-202130). This cross-sectional study adheres to the STROBE statement, which requires written informed consent from healthy controls and patients’ legal representatives.

### 2.2 Clinical assessments

CRS-R evaluations were all performed by experienced neurosurgical clinicians. Subsequently, clinical outcomes encompassing mortality, UWS or MCS from minimally conscious state were obtained through structured telephone interviews with either the patients’ physicians or their relatives, precisely one year following FDG-PET. These outcomes were derived from the six subscales of the CRS-R (23, 24). All CRS-R assessments were performed by trained and experienced clinicians. Clinical outcomes were collected (death, *VS*/UWS, MCS or EMCS) via structured telephone interviews with the patients’ physician or relatives after the FDG-PET within 1 year. This phone interview is based on the six subscales of the CRS-R. Outcomes were grouped in three categories: (1) patients who died, (2) patients who maintained a *VS*/UWS diagnosis or worsened their diagnosis (ie, “poor outcome”), and (3) patients who remained in a MCS or improved their diagnosis such as emerging from MCS (ie, “good outcome”).

### 2.3 FDG-pet

All patients in this study underwent brain 18F-FDG PET/CT using Biograph mCT 52-ring PET/CT manufactured by Siemens AG, Germany, and the contrast agent was 18F-FDG (15). All subjects were asked to fast for at least 6 h. The injected dose was controlled according to the patient’s body mass, and the dose was controlled at 3.7 MBq/kg of 18F-FDG, and then the patients were allowed to rest in a place protected from light for about 1 h. The patient was placed in the supine position, and the brain was scanned using the program provided by the manufacturer, and the time was controlled to be about 3 min. Image reconstruction and post-processing were performed using the manufacturer-supplied program to obtain transverse tomography, coronal tomography, sagittal tomography, and 3-dimensional images. The imaging data were digitally registered to a computer disk to create a tomographic image of the patient’s brain. The distribution of 18F-FDG in the brain was analyzed based on the imaging results, the patient’s brain regions of interest were outlined, and 18F-FDG uptake was observed based on the brain regions of interest (25).

### 2.4 Statistical analysis

Analysis according to clinical research procedures, data were preprocessed and analyzed (15). The data were reoriented and spatially normalized to a stereotaxic space and smoothed with a 14 mm full width Gaussian kernel at half maximum. In order to overcome the problem of big deformations caused by brain lesions as well as the fact that SPM has a default template based on H152O data, we used a customized template described by Phillips et al. for normalization (26). The statistical analyses were conducted using Statistical Parametric Mapping (SPM12^1^). Using the voxel values for each contrast, the statistical parametric map of the *t*-statistics [SPM (23)], was transformed to the unit normal distribution [SPM (23)] and thresholded at voxel-wise 0.05 FDR at the whole-brain level. Statistical analysis of UWS vs. MCS groups we compared the differences between the two groups by SPM12, which in turn gave us the hypometabolic regions.

Using Advanced Normalization Tools (ANTs version 2.0.3), affine and non-linear transformations were employed to register individual images to a common template in Montreal Neurological Institute (MNI) space. This registration process aimed to determine the metabolic index of the best-preserved hemisphere (MIBH). Subsequently, the images were segmented into left and right cortex, and the extracerebral tissue was normalized based on its metabolism. Finally, the metabolic activity was scaled by setting the mean extracerebral activity to a value of 1, with all values ranging from 0 to 1. MIBH was calculated as the highest mean metabolic activity of the two hemispheres. Stender et al. set the cut-off at 3.18 (27). Covariance analysis was used to analyze the effect of age and gender on Mean cortical metabolic index.

### 2.5 Analysis flow chart

**Figure 1:**
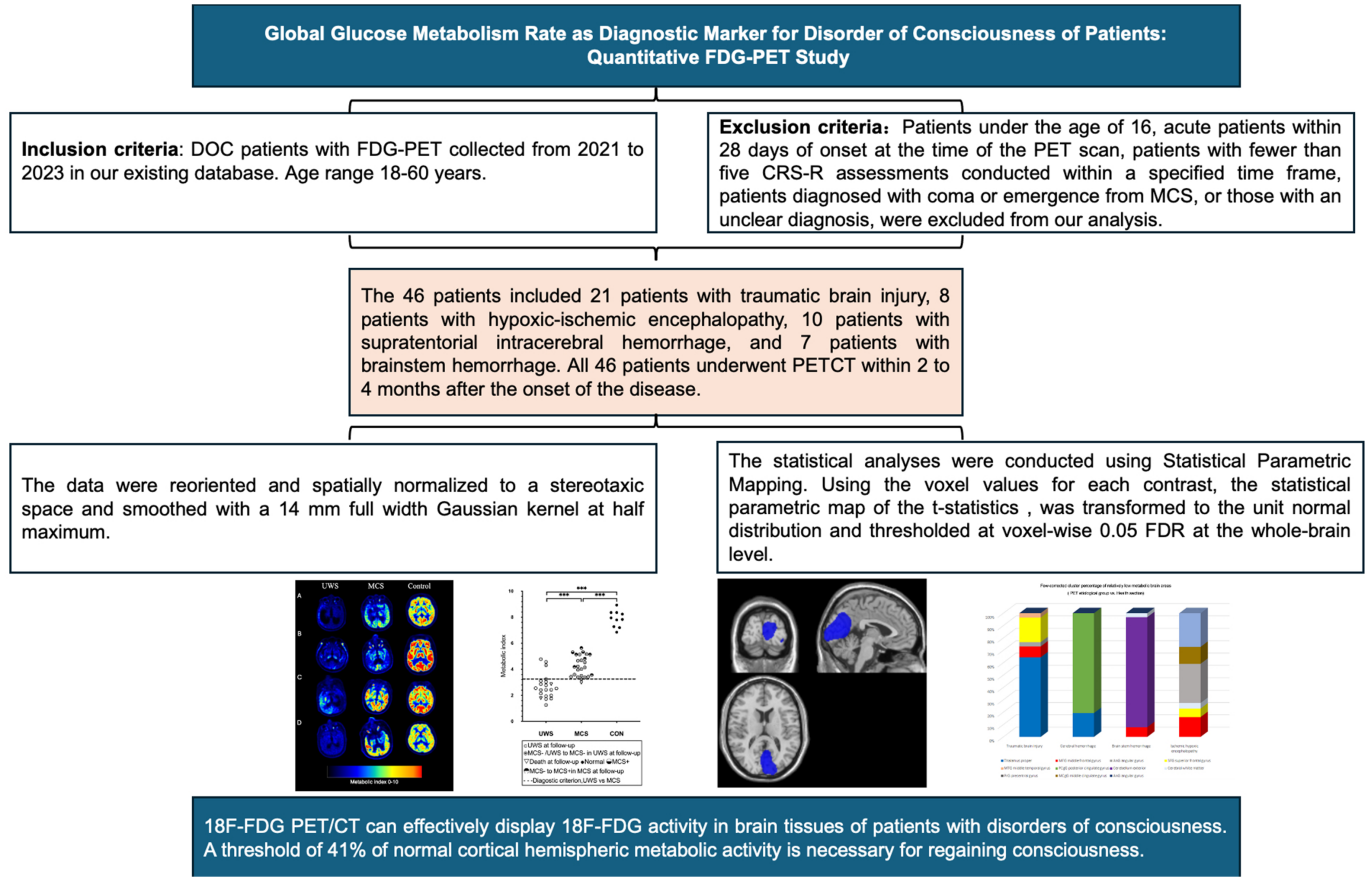
Flow chart of this study.

We followed the flow chart in Figure 1 for data acquisition and further analysis.

## 3 Results

### 3.1 Clinical data

To perform the normalization, a reference distribution was calculated from the extracerebral tissue intensity distributions of the 10 control subjects. Each individual image of the study population was subsequently normalized, by selecting a global scaling factor of the image intensity such that the Jensen-Shannon divergence between the reference distribution and the subject’s extracerebral intensity distribution was minimized (28). We used the mean cortical metabolism to classify between UWS and MCS and predict outcome at 1-year follow-up, using ROC analysis.

### 3.2 Global glucose metabolism rate as diagnostic marker

ANCOVA analysis was used to analyze the effect of age and gender on Mean cortical metabolic index. There was no interaction between age and gender [*F*(1,42) = 0.56, *p* = 0.46] and there was no significant effect of age [*F*(1,42) = 1.72, *p* = 0.20] and gender [*F*(1,42) = 0.81, *p* = 0.37] on Mean cortical metabolic index.

A one-way ANOVA was performed to compare the mean cortical metabolic index of three different group UWS, MCS and healthy controls. A one-way ANOVA revealed that there was a statistically significant difference in cortical metabolic index between at least two groups [*F*(2, 53) = 7.26, *p* < 0.001]. Tukey’s HSD Test for multiple comparisons found that the mean value of cortical metabolic index was significantly different between MCS group (*M* = 4.19, *SD* = 0.64) and UWS group (*M* = 2.74, *SD* = 0.94, *p* < 0.001). Tukey’s HSD Test for multiple comparisons found that the mean value of cortical metabolic index was significantly different between MCS group and healthy group (*M* = 7.88, *SD* = 0.80, *p* < 0.001).

**Figure 2:**
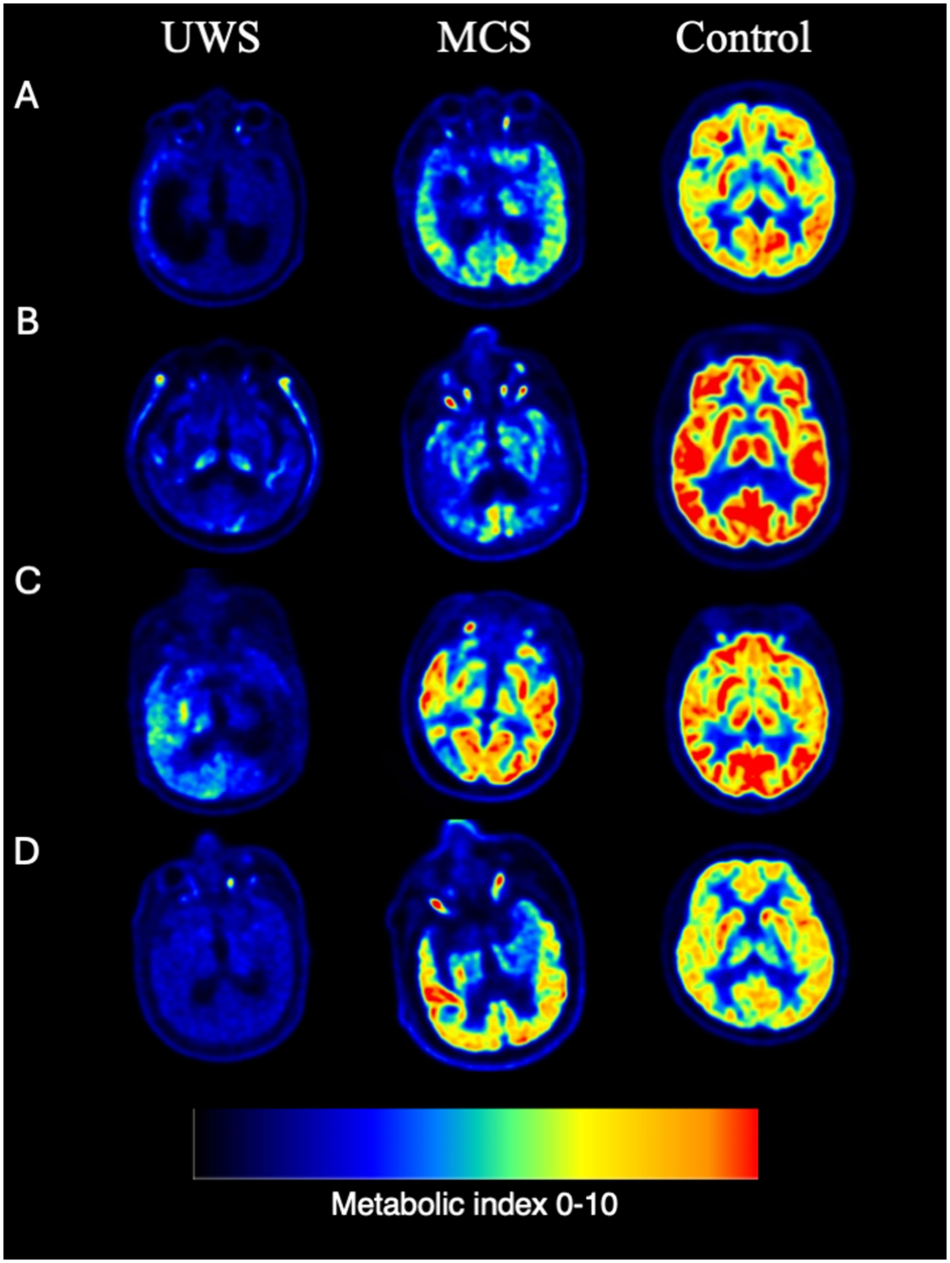
Representative individual FDG PET examples. **(A)** Cerebral trauma. **(B)** Ischemic hypoxic encephalopathy. **(C)** Cerebral hemorrhage. **(D)** Brain stem hemorrhage.

Average cortical glucose metabolism gave diagnostic discrimination between MCS and UWS at an area under the ROC-curve (AUC) of 0.86 (95% CI [0.68–1.0]) in the derivation cohort and 0.93 (95% CI [0.82–1.0]) in the validation cohort. The performance of the classifier did not significantly differ between the two cohorts by ROC Analysis (*p* = 0.64). The best classification rate in the derivation cohort was achieved at a metabolic index of 3.11 (39% of normal activity; Figure 2), resulting in 87% correct classification between UWS and MCS, with 92% sensitivity and 80% specificity to MCS, thus satisfying reasonable requirements for a clinically useful diagnostic criterion. In the validation cohort, this diagnostic threshold provided an 83% correct classification rate between UWS and MCS, with 83% sensitivity and 82% specificity to MCS. *Post hoc* inference suggested an optimal metabolic cutoff at a metabolic index of 3.34 (42% of normal) in the validation cohort; all patient classifications at this level remained unchanged compared to the derivation cohort threshold, i.e., performance of the diagnostic model was similar in the derivation and validation cohorts (Table 2).

**Figure 3:**
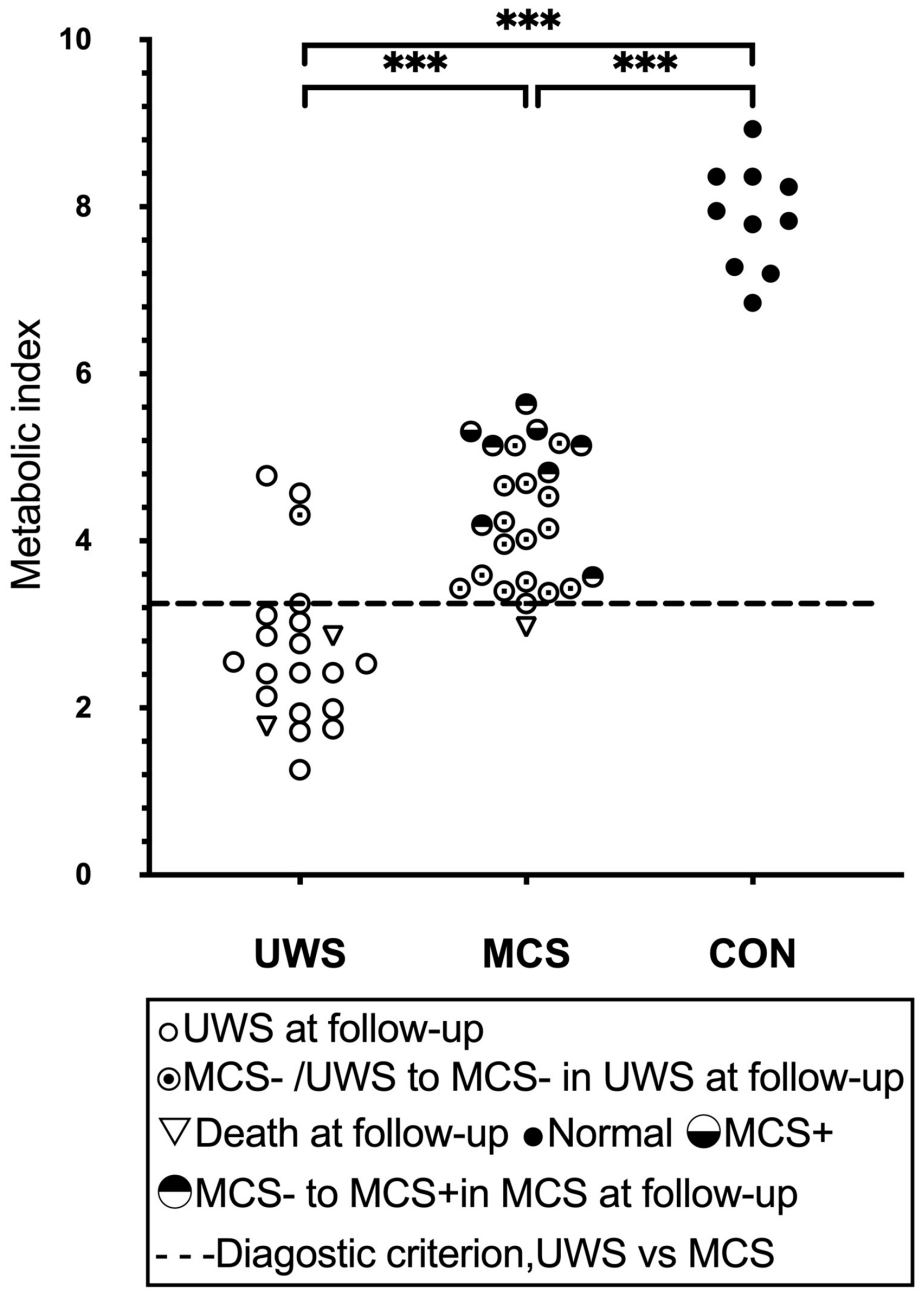
Global brain glucose metabolism. The distribution of quantified cortical metabolic values for all individual subjects in the pooled cohort, with emphasis on the best-preserved hemisphere, is depicted. A dashed line denotes the optimal diagnostic cutoff distinguishing patients in a vegetative state (UWS) from those in a minimally conscious state (MCS). Horizontal lines represent group averages. Metabolic values are expressed as a unitless index, with the average activity in the scaling reference area (extracerebral tissue) standardized to 1. MCS+, high-level behavioral responses; MCS-, low-level behavioral responses.

**Table 2:** Diagnostic threshold for VS and MCS.

Cohort	AUC	Highest classification AUC rate	Cutoff
Derivation	0.862[CI]0.68–1.0	0.87	3.11
Validation	0.932[CI]0.82–1.0	0.826	3.34
Pooled	0.889[CI]0.79–0.99	0.848	3.32

The diagnostic criterion outlined above yielded a diagnostic accuracy of 0.89 (95% CI [0.79–0.99]) as measured by the AUC across the combined cohort. The classification accuracy between MCS and UWS was 85%, with sensitivity and specificity rates of 88 and 81%, respectively, for MCS. The optimal metabolic cutoff point, determined to be a metabolic index of 3.32 (equivalent to 41% of normal), was identified within the pooled cohort. Furthermore, all healthy controls were correctly identified as conscious.

### 3.3 Outcome prediction

Group discrimination by the metabolic criterion correctly predicted 88% of all known patient outcomes, with 95% sensitivity and 76% specificity to manifest consciousness at follow up (Figure 3). A total of 3 UWS patients had brain metabolism above the 41% diagnostic threshold. Of these, 1 had recovered consciousness at follow-up, 2 UWS patients with sub-threshold metabolism died. 6 MCS-patient with over-threshold metabolism progressed to MCS+ on follow-up. There was statistically significant difference in SUV values between the 6 patients (*M* = 4.75, *SD* = 0.75) who progressed to MCS+ and the 16 patients who maintained MCS-in Independent samples *T*-Test (*M* = 4.03, *SD* = 0.65), *t*(20) = 2.22, *p* = 0.038. No one UWS patient with sub-threshold metabolism progressed to MCS on follow-up. 1 MCS patients with near-threshold metabolism died (Figure 3).

### 3.4 Regional metabolic activity

**Figure 4:**
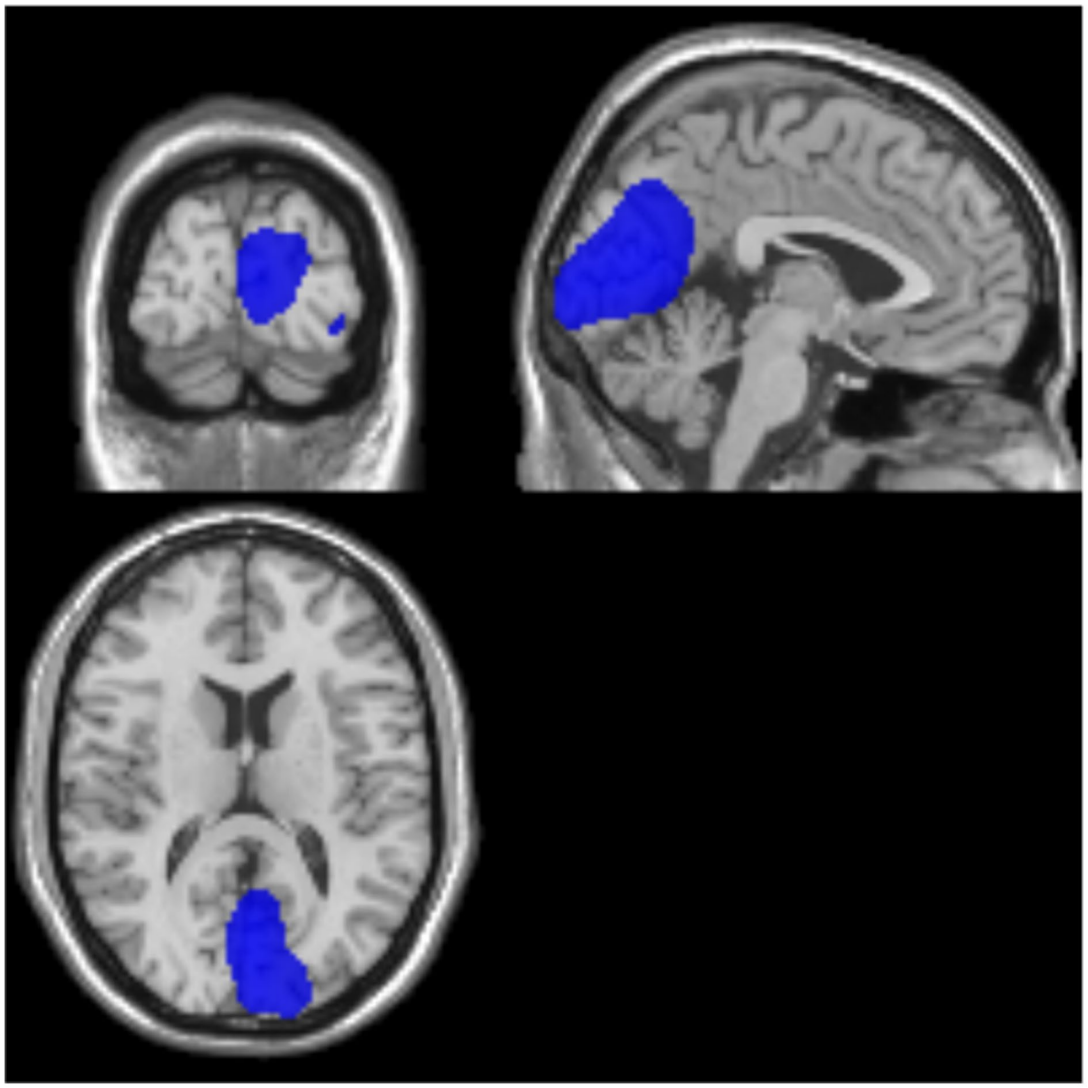
Examples of representative SUV for UWS/MCS. Blue color represents a high brain metabolism. Areas where activity in MCS is significantly higher than in *VS*/UWS (*p* < 0.001). MCS, minimally conscious state; UWS, unresponsive wakefulness syndrome.

The cerebral glucose metabolic rate was generally lower in all patients with impaired consciousness compared to healthy controls, and by comparing and analyzing the cases in the UWS and MCS groups, we found significant differences between the two groups in the left occipital lobe and the left precuneus, with the lower metabolic rate in the UWS group, which may be related to the suggestion of the importance of certain important regions for consciousness (Figure 4).

### 3.5 Regional glucose metabolism

**Figure 5:**
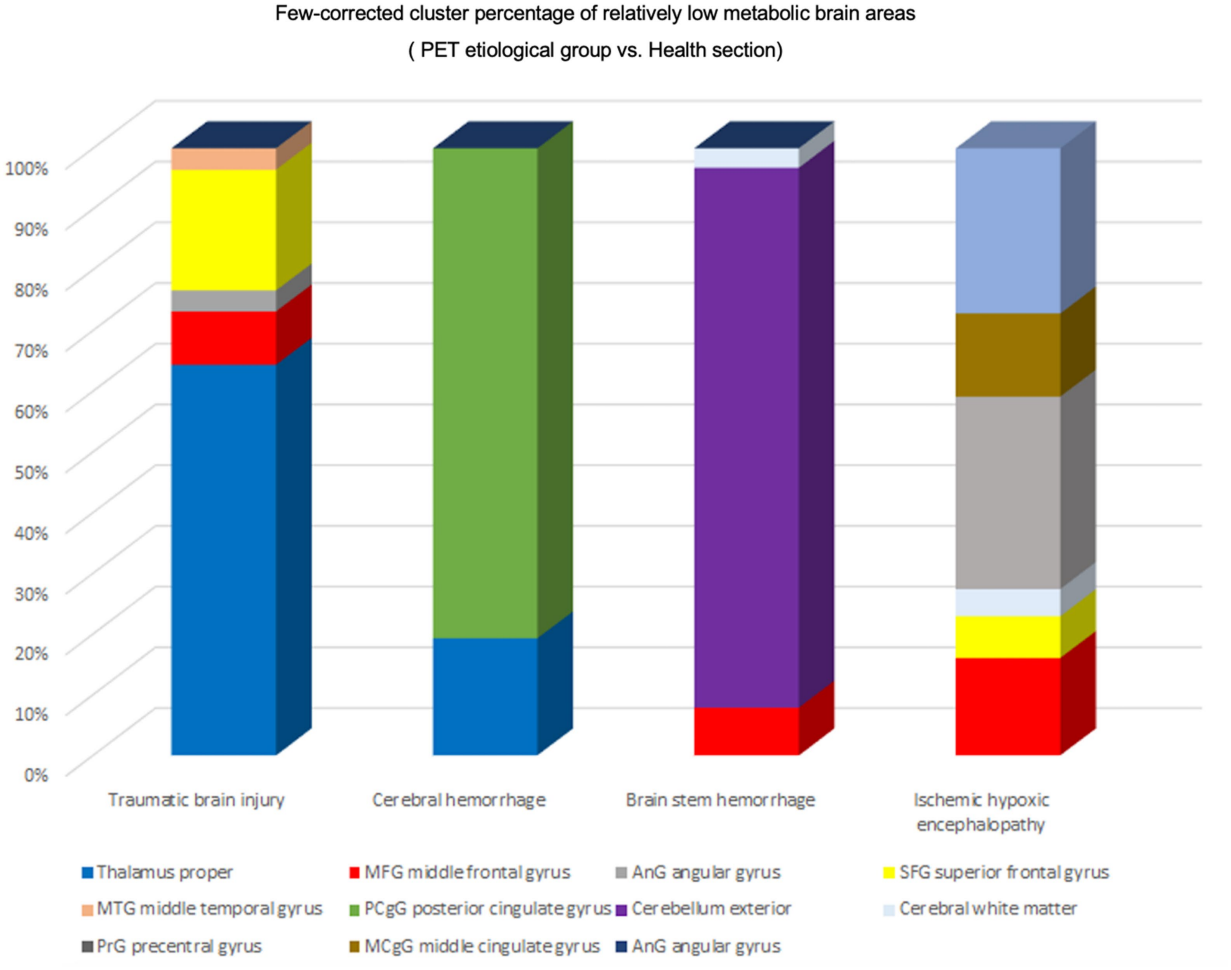
Regional activation of different etiologies. Percentage of Few-corrected clusters of relatively hypometabolic brain regions in the PET etiology group versus the healthy group. TBI Relatively hypometabolic brain regions were predominantly Thalamus proper, Cerebral hemorrhage Relatively hypometabolic brain regions were predominantly posterior cingulate gyrus (PCgG), Brain stem hemorrhage relative hypometabolic brain region is mainly Cerebellum exterior, Ischemic hypoxic encephalopathy relative hypometabolic brain region is mainly angular gyrus (AnG).

The patients’ condition did not exhibit any discernible regional drivers, as metabolic group differences were observed across all volumes of interest (VOIs) (Figure 5). We conducted a comparative analysis of Few-corrected cluster percentages in various disease states to examine hypometabolic brain regions. Our findings indicate that patients with TBI exhibit significant hypometabolism in the thalamus, with subsequent reductions in metabolism observed in the superior frontal gyrus (SFG), middle frontal gyrus (MFG), and angular gyrus. In individuals presenting with cerebral hemorrhage, positron emission tomography (PET) revealed notable hypometabolism in specific brain regions such as the posterior cingulate gyrus, external cerebellum, and thalamus. Conversely, metabolic alterations in other cerebral areas were less pronounced. Conversely, patients with brainstem hemorrhage exhibited reduced metabolism in the cerebellum exterior as well as the middle frontal gyrus (MFG). The observed variations in metabolic activity can be attributed to differing etiologies.

## 4 Discussion

This study illustrates the practicality of quantifying FDG-PET glucose uptake and its effectiveness in accurately diagnosing prolonged DoC. In our study population, we noted that the metabolic threshold that differentiated MCS from UWS was approximately 41% of normal metabolism, a result that aligns with the findings of Stender et al. (27). This inconsistency may be ascribed to protocol-specific variables; nevertheless, our results align with previous studies conducted on individuals with disorders of consciousness, particularly in the context of sleep and anesthesia (29–34). This observation suggests that increased consciousness, starting from the minimally conscious state, may require enhanced activity in particular networks or regions rather than an increase in energy consumption. Additionally, the average cortical glucose metabolic index was determined to be unaffected by etiology or potential confounding variables such as age or gender. Taken together, these findings suggest a significant correlation between cerebral glucose metabolism and the presence of consciousness, irrespective of the etiology of injury or the time elapsed since its occurrence.

Cerebral metabolic activity emerged as a dependable marker for assessing levels of consciousness at the 1-year follow-up. Utilizing a diagnostic threshold of 41% to categorize patients, an 85% accuracy rate was achieved in prognosticating outcomes for the combined cohort of patients with MCS and UWS. This discovery is significant, especially considering the potential limitations in predictive accuracy caused by the elevated mortality rate observed over an extended follow-up period. The model’s precision was evaluated in two distinct cohorts, using behavioral diagnosis and outcomes at a 1-year follow-up as reference points. The results suggest that our assessment of cerebral metabolic activity successfully discriminates between patients in a UWS and those in a MCS, thereby functioning as a dependable diagnostic and prognostic tool for individuals with chronic DoC.

The diagnostic performance of this method was found to be equally effective in both the derivation and validation samples, suggesting its validity across independent cohorts. The ROC analysis of the classification between MCS and UWS revealed a consistent “optimal” diagnostic threshold of 41% of normal metabolic activity, leading to high sensitivity and satisfactory specificity for MCS. Additionally, the method accurately identified healthy controls as conscious individuals. In conclusion, the established FDG-PET diagnostic threshold reliably distinguishes between conscious and unconscious subjects, striking a favorable balance between type I and type II error risks.

In addition, it was found that Occipital Lob and Precuneus hypometabolism may be important points of differentiation. This is consistent with previous studies (19, 35). Precuneus connectivity has demonstrated the ability to effectively distinguish between minimally conscious and unconscious patients, indicating a robust correlation between the activity of this brain region and the patients’ level of consciousness (34, 36). The metabolic level’s specificity in relation to diagnosis and prognosis implies that the classification threshold signifies the minimum energetic demands for sustained consciousness. Our findings notably demonstrate that the shift from unconsciousness to consciousness occurs beyond a clearly delineated global (or hemispheric) metabolic boundary, indicating a global energetic threshold phenomenon that facilitates the restoration of consciousness following brain injury. This discovery aligns with previous assertions that consciousness cannot be solely ascribed to activity in particular networks or regions, but rather is linked to a heightened energetic state encompassing the entire brain. Consequently, the maintenance of glucose metabolic rate in at least one hemisphere emerges as an essential prerequisite (though not the sole determinant) for the restoration of awareness following brain injury. In aggregate, the cortical metabolic level accounted for the present condition or impending recuperation in 94% of patients diagnosed with UWS and MCS.

Clinical studies have shown that 18F-FDG PET/CT can effectively visualize brain cell metabolism and accurately reflect the functional state of the brain. The recovery of consciousness in patients with disorders of consciousness is often mainly transformed from a vegetative state to a minimally conscious state through the neuronal activities of various tissues in the brain to form a complete neural system. The recovery of the higher central nervous system cannot be separated from the neuronal movements in the brain, and all brain tissue activities need to be labeled by glucose metabolism. In this study, we quantified 18F-FDG uptake in DoC patients as a diagnostic index, and used data from a single-center study for statistical analysis, but a multi-center statistical study is more important for reference. In this study, a single modality study was taken, and then, a multimodal study is more helpful for the comprehensive analysis and evaluation of doc patients, such as pet-eeg, which has more important reference value for EEG-metabolic coupling, which will be the direction of future research (15, 37, 38).

## 5 Limitations

Although this study presents an overview of PET imaging’s application in diagnosing DoC and achieves a quantitative analysis of DoC patients, several limitations remain. First, a primary limitation is the relatively small sample size of DoC patients included. Due to the challenges of recruiting patients with stable yet specific levels of consciousness impairment, alongside the high costs associated with PET imaging, patient enrollment was limited. Consequently, our findings may lack broad generalizability. Expanding the sample size in future studies could help enhance the robustness of our results and improve applicability across diverse DoC population sent face-to-face clinical assessment of consciousness levels across all patients proved challenging (39). CRS-R, commonly used to evaluate consciousness, relies significantly on the clinician’s experience and subjective interpretation, which can vary. In-hospital follow-up assessments often provide valuable insights that complement imaging findings, especially when distinguishing between vegetative states and minimally conscious states. Our dependence le clinical records introduces variability in the interpretation of consciousness levels and potentially affects the reliability of these assessments. Additionally, the retroature of data collection introduces a risk of selection bias, which may influence the composition of the patient cohort (40). As this study is a single-center investigation, the patient sample may not represent the broader DoC population, given that it includes only patients for whom PET imaging was clinically indicated. This potential bias may impact the external validity of our findings and restricts the generalizability of our conclusions to a wider DoC population. Lastly, it is essential to ackno complexity of consciousness as a multidimensional construct. While PET imaging provides valuable insights into metabolic activity patterns associated with DoC, it may not capture all facets of consciousness, such as structural connectivity and electrophysiological properties, which are critical for a comprehensive assessment of DoC (33). Thus, PET imaging should ideally be integrother diagnostic tools to ensure a thorough evaluation, as multimodal imaging approaches may offer a more holistic view of consciousness. These limitations highlight the need for continuation, ideally with multicenter collaborations and the integration of additional diagnostic modalities, to enhance our understanding of PET imaging’s role in DoC diagnosis (41).

## 6 Conclusion

In conclusion, 18F-FDG PET/CT can effectively display 18F-FDG activity in brain tissues of patients with disorders of consciousness, which is of positive significance for the assessment of brain function and is worthwhile to be promoted for use in clinical practice. The preservation of hemispheric brain glucose metabolism is indicative of the presence or potential recovery of consciousness in patients with brain injuries. Specific cognitive and sensory functions are supported by regions with preserved metabolic activity. A threshold of 41% of normal cortical hemispheric metabolic activity is necessary for regaining consciousness. FDG-PET serves as a reliable diagnostic and prognostic tool for disorders of consciousness resulting from brain injuries, regardless of their underlying causes.

## Data Availability

The original contributions presented in the study are included in the article/supplementary material, further inquiries can be directed to the corresponding authors.

## Footnotes

^1^ www.fil.Ion.ucl.ac.uk/spm
